# Health beliefs as a key determinant of intent to use anabolic-androgenic steroids (AAS) among high-school football players: implications for prevention

**DOI:** 10.1080/02673843.2017.1344928

**Published:** 2017-07-05

**Authors:** Amanda E. Halliburton, Matthew S. Fritz

**Affiliations:** a Department of Psychology, Virginia Tech, Blacksburg, VA, USA; b Department of Educational Psychology, University of Nebraska-Lincoln, Lincoln, NE, USA

**Keywords:** Steroids, mediation, adolescents, prevention, health behaviour

## Abstract

The use of anabolic-androgenic steroids (AAS) is problematic for youth because of negative effects such as reduced fertility, increased aggression and exposure to toxic chemicals. An effective programme for addressing this problem is Adolescents Training and Learning to Avoid Steroids (ATLAS). This secondary analysis expands prior research by identifying prominent mechanisms of change and highlighting key longitudinal processes that contributed to the success of ATLAS. The current sample consists of high-school football players (*N* = 1.068; *M*_age_ = 15.25) who began ATLAS in grades nine through eleven and participated in booster sessions for two years post-baseline. Knowledge of AAS effects, belief in media ads, reasons not to use AAS, perceived severity of and susceptibility to AAS effects and ability to resist drug offers were critical mediators of the relations between ATLAS and outcomes. Modern applications of the ATLAS programme are also discussed.

The lifetime prevalence of anabolic-androgenic steroid (AAS) use by American high-school males nationwide has increased since 1991, up to 4.0% in 2013 (Centers for Disease Control and Prevention, 2014). AAS are derived from synthetic testosterone (Denham, 2011) and have been associated with positive outcomes (e.g. increased body satisfaction; Kindlundh, Isacson, Berglund, & Nyberg, 1999) but also negative side effects including reduced fertility, gynaecomastia, aggression, depression and suicidality (Irving, Wall, Neumark-Sztainer, & Story, 2002; Lumia & McGinnis, 2010; Petrocelli, Oberweis, & Petrocelli, 2008). Personal trainers and amateur bodybuilders at local gyms may pressure young athletes to try AAS, and they can also be purchased on the Internet. In some cases, toxic fillers or substitute chemicals (e.g. upholstery cleaner) may be added without users’ knowledge (Denham, 2009). Misinformation (e.g. minimizing potential side effects and exaggerating benefits of AAS use) is easily perpetuated through AAS sales, and online buyers in particular can access AAS discreetly and without a prescription (Cordaro, Lombardo, & Cosentino, 2011). Therefore, it is important to understand why adolescents use AAS, despite their negative side effects, instead of changing their diets or focusing on improving their strength-training abilities.

## The ATLAS programme

The Adolescents Training and Learning to Avoid Steroids (ATLAS) programme (Goldberg, Elliot, Clarke, MacKinnon, Moe, et al., 1996; Goldberg, Elliot, Clarke, MacKinnon, Zoref, et al., 1996) is an intervention for high-school football players that focused on preventing AAS and other drug use by presenting healthy alternatives that aligned with team goals. Specific factors that mediated the effect of ATLAS on the outcomes (intent to use AAS, healthy nutrition behaviours and strength training self-efficacy) were identified and targeted based on three prominent health behaviour theories: the Health Belief Model, Social Learning Theory and the Theory of Planned Behaviour. The Health Belief Model (HBM; Janz & Becker, 1984) postulates that a person’s perceptions about the severity of a health condition and susceptibility to its effects, the benefit of trying to prevent the condition and the barriers to taking action all impact health behaviour decisions. According to the HBM, the decision to use AAS is based on an individual’s knowledge about positive and negative effects of AAS, norms of use, perceived severity and susceptibility to adverse effects of AAS use, and ability to refuse drug offers.

Social Learning Theory (SLT; Bandura, 1977) states that people learn by directly experiencing a behaviour or observing others performing the behaviour. According to SLT, knowing about consequences is also critical for planning future behaviours. Thus, SLT emphasizes that others (e.g. coaches, teammates, the media) can reinforce or discourage AAS use and that adolescents who see others benefiting may be more likely to use AAS (Strecher, DeVellis, Becker, & Rosenstock, 1986). Social media facilitates selective observation of the benefits of AAS use because users are unlikely to post about negative side effects, which can be embarrassing (e.g. gynaecomastia). It also provides a platform for connecting with other AAS users across the world. Because teammates go through similar experiences and struggles, student athletes may view teammates as more credible sources of information about health (including nutrition, exercise, AAS use) than teachers or counsellors, and take their opinions seriously.

The Theory of Planned Behaviour (TPB; Azjen, 1991) posits that intention and perceived ability to perform a behaviour, as influenced by attitude toward the behavior and perceived norms, predict future behaviour performance. This theory has been supported in prior studies of doping intentions (Lazuras, Barkoukis, Rodafinos, & Tzorbatzoudis, 2010) and use (Lucidi et al., 2008) among European athletes and students. TPB suggests that intent to use AAS predicts future AAS use and an individual’s intent is based on attitudes about AAS and its users, social norms and perceived ability to refuse offers of AAS (MacKinnon et al., 2001). Individual beliefs and persuasion related to AAS affect not only intent to use AAS but also decisions about alternative behaviours, such as management of a healthy diet and strength training skill building.

### Prior mediation analyses

Drawing on the HBM, SLT and TPB, MacKinnon et al. (2001) evaluated 12 mediators targeted by ATLAS on intent to use AAS (see Table 1) and found significant effects for: knowledge of the effects of AAS, ability to turn down offers of drugs, perceived severity of AAS use, perceived susceptibility to the effects of AAS, reasons for using AAS and reasons for not using AAS. MacKinnon and colleagues also examined, whether these variables mediated the healthier alternatives to AAS use presented in the programme: nutrition behaviours and strength training self-efficacy. The significant mediators of the programmes effect on nutrition behaviours were: team as an information source, peers as an information source and belief in media advertisements. The significant mediators of strength training self-efficacy included knowledge of AAS effects, perceived coach tolerance of AAS use, team as an information source, peers as an information source, ability to turn down drug offers, perceived severity of AAS use, perceived susceptibility to AAS effects and reasons for using AAS.

### Purpose of the present study

Though isolated effects of the ATLAS programme mediators have been examined (MacKinnon et al., 2001), longitudinal relations among these mediating variables have not been explored. Investigating linkages among these variables would assist in revealing specific paths that yield the most notable influences on intent to use AAS. For example, although ATLAS was based on the HBM, SLT and the TPB, these theories may not have contributed equally to key ATLAS processes. Establishing the longitudinal structure of programme variables may help those who intervene with youth at risk for AAS use by clearly identifying the major mechanisms of intervention (i.e. those mediators that change over several time points and help drive the programmes effects) and understanding how programme effects are likely to play out over the long term (which will assist in planning specific intervention targets and goals for participants). Strategies based on this information could be used to strengthen long-term prevention effects, reduce the likelihood of relapse for former AAS users who enter the programme, and help interventionists make the most of limited financial and other resources in their local schools.

**Figure 1:**
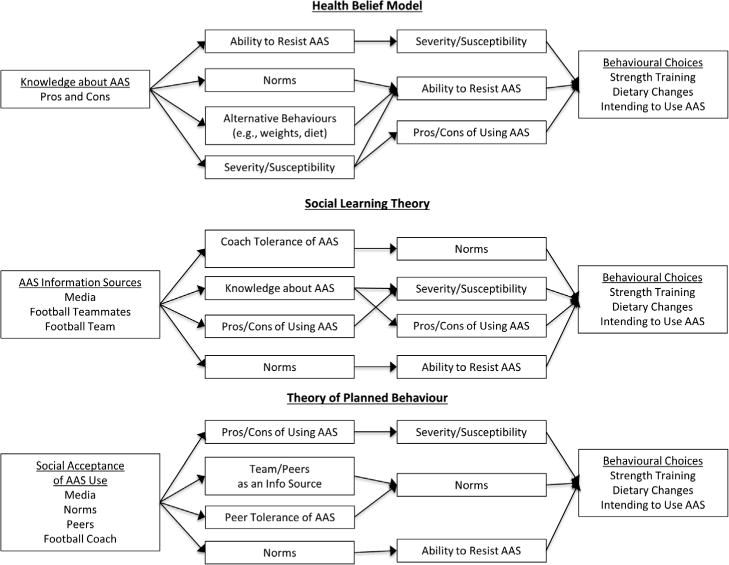
Conceptual model of AAS use based on the three models utilized in the ATLAs programme (Health Belief Model, Social Learning Theory and Theory of Planned Behaviour).

In order to construct a longitudinal mediation model of ATLAS and evaluate, whether the HBM, SLT and the TPB contributed equally to its key processes, the mediators and outcomes in Table 1 must first be ordered according to the HBM, SLT and TPB as illustrated in Figure 1. According to the HBM, knowledge about AAS, specifically pros and cons of use, is the most direct effect of the ATLAS programme. AAS knowledge affects perceived ability to resist using AAS, how common AAS use is perceived to be (norms), how severe the consequences of AAS use are likely to be, and how susceptible someone may be to those consequences. In addition, AAS knowledge informs the performance of nutrition behaviours and awareness of strength training self-efficacy (i.e. in comparing various strategies to improve physique and athletic ability). The ability to resist AAS also impacts perceived severity and susceptibility because if someone is prone to substance use, they are likely not as concerned about the severity of the consequences or susceptibility to those consequences. Norms, skills and knowledge related to nutrition and strength training, and perceived severity and susceptibility also impact the ability to resist AAS in the future. Additionally, perceived severity and susceptibility affect knowledge about AAS pros and cons (i.e. if certain consequences are perceived to be more severe or more likely, participants would be more likely to report them as notable pros and cons of AAS use). Perceived severity and susceptibility, the ability to resist AAS, and pros/cons of using AAS affect AAS intent, strength training self-efficacy and nutrition behaviours.

Using SLT, the most salient direct effect of ATLAS is increased awareness of and attention to social sources of information about AAS (the media, football teammates, knowledge and experience gained from being part of the football team). These information sources affect perceived tolerance of AAS use by the coach, as well as participants’ knowledge of AAS, the pros and cons of AAS use, and AAS use norms (particularly among other members of the football team). Perceived coach tolerance affects AAS norms because if the coach is perceived to be less tolerant of AAS, the players are less likely to use AAS (and vice versa). Knowledge about AAS affects perceived severity and susceptibility and the pros and cons of AAS use. The pros and cons of AAS use also affect perceived severity of and perceived susceptibility to those effects. Norms affect the perceived ability to resist AAS because it may seem more difficult to resist AAS offers if more people within someone’s immediate social circle are also using AAS. Norms, perceived severity and susceptibility, pros and cons of AAS use, and the ability to resist AAS affect AAS intent, strength training self-efficacy and nutrition behaviours.

Based on the TPB, the most salient direct effect of ATLAS would be information about social acceptance of AAS use based on the media, norms, peers, and the football coach. These beliefs and knowledge affect pros and cons of using AAS (because this is the information most likely to be transmitted to potential users), perception of the team and peers as sources of information about AAS (i.e. whether they provide accurate information), perceived peer tolerance of AAS and norms. Pros and cons of AAS use affect perceived severity of and susceptibility to these consequences. Perception of the team and peers as information sources and perceived peer tolerance affect norms, particularly in the participant’s immediate social circle. Norms affect the ability to resist AAS offers. Severity and susceptibility, norms, and the ability to resist AAS affect intent to use AAS, nutrition behaviours and strength training self-efficacy.

Next, the individual HBM, SLT and TPB models of ATLAS in Figure 1 were combined into a comprehensive longitudinal mediation model. This model not only integrates HBM, SLT and TPB but also includes the timing of the variable measurements, which were selected for analysis based on the hypothesized order of processes in these three theories. The process of refining and modifying this model to improve overall fit is described in the Results section.

## Method

### Design of original ATLAS study

Football players on varsity teams from 34 high schools in the Pacific north-west were recruited for the IRB-approved ATLAS programme. Schools were matched on socio-economic status and win/loss records, then randomized to condition. Three schools dropped out prior to the intervention, resulting in 15 schools in ATLAS and 16 in the control condition. All participating schools received $3000 worth of weight room equipment (Goldberg, Elliot, Clarke, MacKinnon, Zoref, et al., 1996). The experimental schools received 14 sessions scheduled over seven weeks. Seven sessions were devoted to strength training and conducted in the weight room, while the others were delivered in a classroom setting (MacKinnon et al., 2001). Control schools were given a pamphlet about AAS use (Goldberg, Elliot, Clarke, MacKinnon, Moe, et al., 1996).

Data were collected using a self-report questionnaire and anthropomorphic measures (e.g. body fat percentage; MacKinnon et al., 2003). Students in both conditions were measured prior to the start of the football season and immediately after the season. Seniors were measured again in the spring prior to graduation. Non-seniors who remained on the team the following year were measured again before and after the season, during which they received a booster session. Juniors from the first year were measured again in the spring prior to graduating. This progressed for four years. Students who joined the team each year became part of a new cohort (e.g. students who joined the team during the second year of data collection joined the second cohort). Parental consent was obtained prior to enrollment of students in the study.

### Data for present study

Only students in Cohort 1 (*N* = 1.506) were used for the current study, which represents a secondary analysis of the original ATLAS data-set. Seniors from Cohort 1 were removed (*N* = 438) as they did not have data for the second year, resulting in a final sample size of 1068. Students in the final sample were measured prior to the football season to establish a baseline (zero months), after the season ended (three months), immediately prior to the following season (12 months) during which they received a booster intervention, immediately after the second season (15 months), and a final time at either 21 months for students graduating at the end of the second year or 24 months for non-graduating students (time points combined; 24 months). The final sample (*M*_age_ = 15.25; SD = .887) was mostly white (77.8%) and had mothers (67.7%) and fathers (72.7%) with at least a high-school diploma.

### Measures

Table 1 contains basic descriptions of all variables used in the current study; information about the overall questionnaire has been provided elsewhere (Goldberg, Elliot, Clarke, MacKinnon, Moe, et al., 1996; Goldberg, Elliot, Clarke, MacKinnon, Zoref, et al., 1996). Multi-item construct scores were obtained using the mean of the item scores with the following exceptions: knowledge of AAS effects, which was scored on a seven-point Likert scale and then recoded into agree/disagree scores for each item and summed, and reasons to use and not to use AAS, which were based on the sum of reasons endorsed. Descriptive statistics and reliabilities for mediators and outcome variables are displayed in Table 2; standardized alphas were used because constructs varied in their number of items and scales (Falk & Savalei, 2011).

### Analyses

Models were estimated using Mplus (Version 6.12, Muthén & Muthén, 2011). Missing data were handled using full information maximum likelihood (Enders, 2010). Fit criteria included chi-square goodness of fit (*χ*^2^), Bayesian Information Criterion (BIC), Comparative Fit Index (CFI; Hu & Bentler, 1999) and root-mean-square error of approximation (RMSEA). Mediation effects were estimated using MODEL INDIRECT and 95% confidence intervals (CIs) were created using the bias-corrected bootstrap (MacKinnon, 2008) with 1000 resamples. The CLUSTER command in Mplus was initially used to adjust standard errors for nesting of students within schools, but the bootstrapping function cannot be combined with this command. An examination of results with and without the CLUSTER command yielded minimal differences, so these results do not include the CLUSTER command so that bootstrap CIs may be presented.

## Results

### Overall model fit

Initially, a complex path model based on the theorized relations from the HBM, SLT, and TPB (as displayed in Figure 1) was created. The model fit was somewhat poor, with some disagreements between indices (*χ*^2^ [302] = 947.844, *p* < .001; BIC = 85760.031; RMSEA = 0.045; CFI = 0.868). This model was simplified in an attempt to improve fit by removing variables at certain time points that did not contribute substantially to the model (e.g. those that did not relate significantly to at least one outcome variable) and reducing the number of non-significant paths. In total, six variables at two different time points (although they were all represented at other time points in the model) and 38 paths were removed from the model.

**Figure 2:**
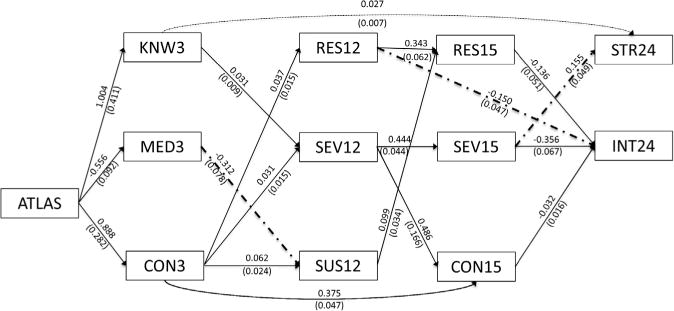
Simplified final longitudinal model of the ATLAS programmes effects, with path coefficients and standard errors. Notes: Variable names correspond to abbreviations in Table 1. Variable numbers correspond to time point (i.e. 0, 3, 12, 15 or 24 months) as shown in Table 2. Also, though not displayed here, all variables occurring at the same time were correlated. Variables measured at 3 months or later were regressed onto prior time points (except baseline; model fit was compared with and without baseline included and no noteworthy differences were observed between the two versions, so baseline measures were omitted) as shown, where applicable. Only the significant indirect effects from the final mediation model are shown to maximize readability of this figure. Dash–dotted lines represent paths added based on modification indices.

The overall fit of the revised model was adequate (*χ*^2^ [186] = 515.384, *p* < .001; BIC = 67340.790; RMSEA = 0.041; CFI = 0.904). Modification indices were evaluated to further improve fit. In order to not over fit the model to this sample, only modifications that could be supported by the HBM, SLT- and/or TPB were considered; the theoretical basis for each of the new paths is described in the discussion. Four new paths were added in the final model; no other changes were made between the initial and final model. These added paths are shown in Figure 2, along with a simplified view of only the significant indirect effects from the revised model. Final fit was good (*χ*^2^ [182] = 450.688, *p* < .001; BIC = 67303.988; RMSEA = 0.037; CFI = 0.922) and significantly better than the unmodified model (*χ*^2^_D_ [4] = 64.696, *p* < .001).

According to the final model (see Figure 2), the ATLAS programme increased knowledge and reasons not to use AAS, while reducing belief in media ads at 3 months. These changes predicted increases in perceived severity and susceptibility, as well as an improved ability to resist at 12 months. Additionally, knowledge was related to an increase in strength training self-efficacy at 24 months. Ability to resist and perceived severity and susceptibility at 12 months predicted increases in ability to resist, reasons not to use and perceived severity at 15 months, which in turn were related to a reduction in intent and an increase in self-efficacy at 24 months. Notably, this figure does not represent a modified version of the revised model; rather, for ease of viewing, this figure depicts only the significant indirect effects from the revised model, along with the modification indices that were added to this model to improve fit, as described above.

### Indirect effects

Though all paths in Figure 2 are significant, MacKinnon (2008) recommends testing for the significance of indirect effects using a confidence interval around each estimate. The confidence intervals used here were created using the bias-corrected bootstrap procedure because it does not assume a normal distribution of effects, typically has higher power than the percentile bootstrap, and has a low risk of elevated Type I error rate in large sample sizes (i.e. > 500, Fritz, Taylor, & MacKinnon, 2012). The seven significant indirect effects are shown in Table 3. Note that no significant indirect effects were found between the ATLAS programme (at zero months) and nutrition behaviours (at 24 months); this finding is revisited in the discussion.

Five significant indirect effects were found between the ATLAS programme (at zero months) and intent to use AAS (at 24 months). ATLAS group status increased the number of reasons not to use AAS at 3 months, which increased ability to resist drug offers at 12 months, which reduced intent to use AAS (*β* = −0.005; SE = 0.003; 95% CI = [−0.015, −0.001]). ATLAS increased reasons not to use AAS at three months, which increased reasons not to use AAS at 15 months, which reduced intent (*β* = −0.011; SE = 0.006; 95% CI = [−0.029, −0.002]). ATLAS also increased knowledge of AAS effects at three months, which increased perceived severity of AAS effects at 12 months, which increased perceived severity at 15 months, which reduced intent (*β* = −0.005; SE = 0.003; 95% CI = [−0.013, −0.001]). ATLAS increased reasons not to use AAS at three months, which increased perceived severity at 12 months, which increased perceived severity at 15 months, which reduced intent (*β* = −0.004; SE = 0.003; 95% CI = [−0.014, −0.001]). Finally, ATLAS reduced belief in media ads at three months, which reduced perceived susceptibility at 12 months, which increased ability to resist at 15 months, which reduced intent (*β* = −0.002; SE = 0.001; 95% CI = [−0.007, −0.001]).

Two significant indirect effects were found between ATLAS and strength training self-efficacy (at 24 months). ATLAS increased knowledge at three months, which increased self-efficacy (*β* = 0.027; SE = 0.013; 95% CI = [0.008, 0.064]). Also, ATLAS increased knowledge at three months, which increased perceived severity at 12 months, which increased perceived severity at 15 months, which increased self-efficacy (*β* = 0.002; SE = 0.001; 95% CI = [0.001, 0.007]).

## Discussion

The ATLAS programme has previously been shown to be successful in reducing intent to use AAS (Goldberg, Elliot, Clarke, MacKinnon, Moe, et al., 1996). The present study investigated the longitudinal processes by which this outcome was achieved. Seven significant indirect effects were found, five that affected intent to use AAS at 24 months and two that affected strength training self-efficacy at 24 months. These relations suggest that the HBM is predominant among the three theories included in this study in facilitating longitudinal effects of ATLAS, although the small indirect effect sizes noted in the present analysis should be noted.

Specifically, perceived susceptibility to AAS effects and reasons not to use AAS influenced perceived ability to resist drug offers, and reasons not to use AAS and perceived severity of AAS effects seemed to evolve over time and affect later intent to use AAS. These suggests that potential users focus on the negative consequences of AAS and how dangerous or likely to occur they are when making decisions about wanting to try AAS. They also consider their ability to resist offers of AAS, which may involve critically evaluating claims about the benefits and consequences of AAS use. Knowledge of AAS effects and perceptions about their severity also influenced perceived strength training self-efficacy, suggesting that they affect the decision to improve weight training abilities as an alternative to AAS use (i.e. potential AAS users decide which option is likely to have the best outcome). Based on this model, the ATLAS programme may be especially important for youth who demonstrate difficulties with thinking through the decision to try AAS or those who place too much confidence in incorrect information about AAS (e.g. as perpetuated by testimonials that are shared by other athletes).

These findings partly concur with recent updates to the HBM, which suggest that perceived severity of and susceptibility to potential health problems may not yield direct effects on health outcomes but are instead likely to yield indirect effects via benefits of and barriers to health changes, as well as self-efficacy to make those changes (Carpenter, 2010). However, this model still supports the relevance of severity and susceptibility to AAS intent. This may be due to the fact that AAS are somewhat unknown to many youths compared to more commonly used substances, and the severity of their effects, along with individual response differences, may be underestimated. Also in agreement with Carpenter (2010), severity of AAS effects figured more prominently and with clearer longitudinal effects in our model than did susceptibility to effects.

Additionally, as suggested by SLT and/or TPB, responses to perceived susceptibility (e.g. resistance, intent) were also impacted by changes in belief in media ads during the programme. The path between belief in media advertisements and perceived susceptibility was added to the final model based on modification indices, and it was not predicted by the theoretical models pictured in Figure 1. However, this finding indicates that the media is an important source of information about AAS effects and norms and may put potential AAS users in danger by providing misinformation (e.g. by marrying AAS with images of popular, successful athletes and downplaying the likelihood of negative effects). As demonstrated by the final model, ATLAS successfully challenged some of this misinformation, and the skills taught by ATLAS may assist in reducing belief in media advertisements and subsequently reducing AAS intent by causing participants to reconsider the perception that harmful effects are unlikely.

Unexpectedly, no significant indirect effects were found between the ATLAS programme and nutrition behaviours in this study. Based on the best fitting model used here, this finding suggests that indirect processes other than those facilitated by perceived severity of and susceptibility to AAS effects, ability to resist AAS, and reasons not to use AAS may affect nutrition behaviours, in contrast to intent to use AAS and strength training self-efficacy. Notably, the final model only included one of the mediators that were significant for nutrition behaviours in the original mediation analysis (MacKinnon et al., 2001). Other models of ATLAS that utilize the HBM, SLT and TPB and ATLAS programme variables differently may reveal these processes.

### Strengths and limitations

The use of a longitudinal design and inclusion of a strong theoretical framework are the major strengths of this study, although not all variables were available at all time points given the structure of the data. The major weakness of the study is that we utilized secondary data analysis, which necessitates a data-driven model. In addition, intent to use AAS was used in place of actual AAS use as a major outcome variable because actual AAS use was reported by few participants and in small quantities, and intent to use AAS demonstrated a less restricted range of responses. Given the young age of the ATLAS participants, however, intent to use AAS may be a more relevant variable than actual AAS use, particularly from a primary prevention standpoint. Additionally, a potential weakness of the original programme design is that schools that received the intervention received more hands-on training than control schools, which may potentially have positively influenced the success of the programme for those schools that received it.

### Implications for prevention

AAS use differs in several ways from the use of other substances and more research and specified preventative efforts for AAS should be generated. However, aside from ATLAS, few programs for AAS have been developed and peer-reviewed (Kuehn, 2009). The present study suggests that AAS prevention may be implemented using a multi-faceted approach: providing accurate information about AAS, addressing popular misconceptions about AAS, teaching skills to help participants resist offers of AAS, allowing participants to evaluate their own perceptions about AAS use, and discussing healthier alternatives to AAS use for improving body image and performance. Findings from the original ATLAS papers and the salient components identified herein may be useful for creating and structuring other prevention programs for youth AAS use.

Since ATLAS was first implemented, the characteristics of AAS use have changed in several ways. Discussion of performance-enhancing substances, including AAS, has become more commonplace with emerging news stories of state-sponsored doping programmes at the 2016 Summer Olympics, along with recent testimonies of AAS use by athletes in the Major Baseball League (MLB) and Tour de France, among other sports leagues and organizations (Momaya, Fawal, & Estes, 2015). The use of these substances has expanded beyond professional athletes, however, and spread more widely to young athletes (like those who participated in the present study), gym members concerned with their physical image, and prisoners (Sjöqvist, Garle, & Rane, 2008). AAS are now widely available over the Internet and can be easily and discreetly obtained, often without much questioning as to whether a physician has authorized the use of AAS; this avenue of purchase may be particularly preferred by youth who are concerned about the social stigma related to buying AAS (Clement, Marlowe, Patapis, Festinger, & Forman, 2012). While, Internet purchases of AAS are convenient, misinformation about the benefits and dangers of AAS use may also be easily disseminated online (Clement et al., 2012). The rise of social media has exacerbated both of these problems by connecting potential AAS users with sellers around the world and allowing for the mixing of facts with dangerous myths. Exposure to sports-, fitness- and body image-related media, which is facilitated by the user-friendly, wide-spread connectivity of social media, has been found to predict AAS use in European youth (Frison, Vandenbosch, & Eggermont, 2013) and more research on the influence of modern technology on adolescent AAS use needs to be conducted with participants from the US. Additionally, as the theories that first inspired the development of the ATLAS programme have evolved, the programme itself would also benefit from evolving further to match them more closely.

Despite the passage of time, however, the ATLAS programme remains very relevant for youth growing up in the current climate of AAS use. One of the programmes key strengths is its focus on both positive and negative aspects of AAS use, and this dual focus allows facilitators to effectively address misinformation about AAS taken from the Internet or social media. As part of the ATLAS curriculum, participants discuss media myths and claims about AAS effects and refer to advertisements containing professional athletes and celebrities as examples. Performance-enhancing substance use in prominent sports leagues and organizations are good examples to use in these discussions because they are recent and likely to generate comments. Based on the importance of HBM in the results of the present study, these types of modules serve an important role in reducing intent to use AAS and promoting healthy alternative behaviours. Additionally, ATLAS and similar programmes can help call adolescents’ attention to real-life models of sports performance, particularly if teammates and coaches can speak personally to their negative experiences with or exposure to AAS use and its dangerous consequences.

These findings, which need to be replicated, suggest that an increase in knowledge of AAS, reduced belief in advertisements, and an increase in reasons not to use AAS are the most direct effects of ATLAS. The programme dispels potential myths about advertisements related to AAS and also provides accurate information about the benefits and consequences of AAS use. Skills training for resisting AAS and discussions about perceived severity and susceptibility to negative consequences of AAS use are also important. AAS use reduction is a good target for further research, and the ATLAS programme provides a good template for the development of new AAS use prevention programmes, particularly if the components highlighted in this study are included. In keeping with the HBM, future research may wish to augment findings from ATLAS by exploring the extent to which youth perceive benefits of and barriers to engaging in alternatives to AAS use, in order to bolster preventative effects of programmes for reducing intent to use AAS. Additionally, as mentioned above, more information about the connection between modern technology (particularly the Internet and social media) and youth AAS use would be beneficial for shaping future applications of ATLAS and similar interventions.

## Figures and Tables

**Table 1:** ATLAS constructs: mediators and outcome variables.

Mediators	Variable name	Rating scale	Sample item
Perceived coach tolerance of AAS use	CCH	1–7	I have talked with my coaches about alternatives to AAS use
Reasons for not using AAS	CON	0–14	‘Afraid of becoming addicted,’ ‘because it is cheating,’ etc.
Knowledge of the effects of AAS	KNW	0–18	‘Improve physically,’ ‘more arguments and fights,’ etc.
Belief in media advertisements	MED	1–7	Products advertised in muscle magazines do what they claim
Normative beliefs about AAS use	NRM	0–11	Out of every 100 HS football players at your school, how many do you think have ever used AAS, even once?
Peers as an information source	PER	1–7	Team leaders teach me about drug prevention
Perceived peer tolerance of AAS use	PTL	1–7	My teammates don’t care if I use AAS
Reasons for using AAS	PRO	0–9	‘Get stronger,’ ‘become a better athlete,’ etc.
Ability to turn down offers of drugs	RES	1–7	I could turn down a weight lifter offering me AAS
Perceived severity of AAS Use	SEV	1–7	The bad effects of AAS go away when you stop using them
Perceived susceptibility to the effects of AAS	SUS	1–7	I would have no bad side effects from using AAS
Team as an information source	TEM	1–7	Being on the football team has improved my health

Outcome variables	Variable name	Rating scale	Sample item

Intent to use AAS	INT	1–7	I intend to use AAS
Nutrition behaviours	NUT	1–7	My diet has less than 30% of calories from fat
Strength training self-efficacy	STR	1–7	I know how to train with weights to become stronger

Note: All scales are constructed such that a higher number reflects a greater or higher amount of the construct.

**Table 2:** Descriptive statistics for mediators and outcome variables at each time point.

	No. of items	0 months		3 months		12 months		15 months		21 months (seniors)		24 months	
	
Construct		*α*	*M* (SD)	*α*	*M* (SD)	*α*	*M* (SD)	*α*	*M* (SD)	*α*	*M* (SD)	*α*	*M* (SD)
CCH	3	.642	2.14(1.23)	.780	2.10(1.33)	.769	2.09(1.37)	.790	2.20 (1.44)	.778	2.12(1.27)	.693	2.22 (1.36)
CON	14	.846	6.89 (3.53)	.887	7.28 (4.02)	.900	7.13(4.19)	.891	6.63(4.11)	.875	5.82 (3.85)	.907	6.92 (4.35)
INT	5	.919	1.72(1.26)	.920	1.71 (1.15)	.917	1.72 (1.22)	.922	1.74(1.21)	.928	1.80(1.19)	.956	1.89(1.41)
KNW	18	.895	10.21 (4.53)	.921	11.77 (4.99)	.923	11.66 (4.78)	.942	11.79 (4.90)	.948	11.86 (4.92)	.955	11.20 (5.30)
MED	3	.750	2.81 (1.26)	.800	2.57(1.29)	.812	2.65(1.35)	.847	2.50(1.41)	.817	2.40 (1.27)	.831	2.70(1.35)
NRM	3	.824	2.51 (1.66)	.796	2.23 (1.55)	.820	2.30(1.61)	.855	1.90 (1.44)	.835	1.90 (1.38)	.812	2.11 (1.62)
NUT	7	.809	3.98(1.13)	.818	4.19(1.09)	.805	4.08 (1.12)	.814	4.15(1.16)	.881	4.12(1.30)	.831	4.05 (1.20)
PER	3	.844	4.48 (1.54)	.885	4.91 (1.60)	.863	4.86 (1.59)	.884	5.11 (1.58)	.893	5.12(1.67)	.872	4.96 (1.47)
PTL	5	.921	2.99 (1.79)	.923	3.04 (1.82)	.900	3.40 (1.86)	.892	3.35(1.82)	.854	3.84 (1.69)	.897	3.70 (1.86)
PRO	9	.879	1.44 (2.29)	.866	1.15(2.06)	.883	0.94 (1.92)	.848	0.78(1.71)	.906	0.68 (1.79)	.830	2.34(2.30)
RES	4	.882	5.96 (1.39)	.897	5.96(1.35)	.867	5.97 (1.36)	.911	5.90 (1.47)	.933	6.20(1.22)	.918	5.92 (1.48)
SEV	3	.814	5.80 (1.26)	.822	5.89 (1.26)	.827	5.74(1.30)	.831	5.79(1.28)	.831	5.80 (1.24)	.842	5.75(1.38)
SUS	3	.713	6.07(2.12)	.757	6.13(2.23)	.760	6.04 (2.20)	.772	5.92 (2.33)	.815	5.78 (2.27)	.753	6.13(2.28)
STR	6	.883	5.64(1.15)	.900	5.85 (1.07)	.919	5.91 (1.16)	.906	5.88(1.15)	.908	6.10(1.06)	.921	5.90(1.16)
TEM	3	.769	5.53(1.13)	.838	5.66(1.20)	.801	5.74(1.17)	.815	5.63 (1.29)	.772	5.82(1.14)	.823	5.70(1.24)

Notes:Three variables had significant differences between the control and experimental groups at baseline: MED (*t* [1036] = 2.82; *p* = .0049), PER (*t* [1043] = 2.11; *p* = .0350), and STR (*t* [983] = 3.69; *p* = .0002). In all three cases, the control group had a higher score than the experimental group. In the original study, ATLAS participants achieved significant change in the expected directions on all three variables compared to the control group (Goldberg, Elliot, Clarke, MacKinnon, Moe, et al., 1996). Differences may be due to the fact that schools were assigned to condition but students were analyzed individually.

**Table 3:** significant total and indirect effects in final model.

Effect	Unstandardized coefficient (standard error)	95% confidence interval for unstandardized coefficient	Standardized coefficient (standard error)
Total effect on intent to use AAS (IN24)	−0.028 (0.013)	(−0.058, −0.006)	−0.011 (0.005)
ATLAS→CON3→RES12→INT24	−0.005 (0.003)	(−0.015, −0.001)	−0.002 (0.001)
ATLAS→CON3→CON15→INT24	−0.011 (0.006)	(−0.029, −0.002)	−0.004 (0.002)
ATLAS→KNW3→SEV12→SEV15→INT24	−0.005 (0.003)	(−0.013, −0.001)	−0.002 (0.001)
ATLAS→CON3→SEV12→SEV15→INT24	−0.004 (0.003)	(−0.014, −0.001)	−0.002 (0.001)
ATLAS→MED3→SUS12→RES15→INT24	−0.002 (0.001)	(−0.007, −0.001)	−0.001 (0.001)
Total effect on strength training self-efficacy (STR24)	0.040 (0.015)	(0.012, 0.074)	0.018 (0.007)
ATLAS→KNW3→STR24	0.027 (0.013)	(0.008, 0.064)	0.012 (0.006)
ATLAS→KNW3→SEV12→SEV15→STR24	0.002 (0.001)	(0.001, 0.007)	0.001 (0.001)

Note: 95% confidence intervals were created using the bias-corrected bootstrap.
